# Excessive ER-phagy mediated by FAM134B contributes to trophoblast cell mitochondrial dysfunction in preeclampsia: Excessive ER-phagy causes mitochondrial apoptosis in preeclampsia trophoblast

**DOI:** 10.3724/abbs.2024065

**Published:** 2024-05-22

**Authors:** Andi Wang, Zhuo Li, Dan Zhang, Chang Chen, Hua Zhang

**Affiliations:** 1 Department of Obstetrics and Gynecology the First Affiliated Hospital of Chongqing Medical University Chongqing 400016 China; 2 The Chongqing Key Laboratory of Translational Medicine in Major Metabolic Diseases the First Affiliated Hospital of Chongqing Medical University Chongqing 400016 China; 3 Institute of Life Sciences Chongqing Medical University Chongqing 400016 China

**Keywords:** preeclampsia, endoplasmic reticulum autophagy (ER-phagy), mitochondria-associated endoplasmic reticulum membrane, mitochondrial dysfunction, lipidomic metabolome

## Abstract

Autophagy dysregulation and Ca ^2+^-induced mitochondrial dysfunction in trophoblast cells are proposed to contribute to preeclampsia (PE) development. FAM134B is identified as a receptor associated with endoplasmic reticulum autophagy (ER-phagy). In this study, the placentas of normal pregnant women and PE patients are collected and analyzed by immunohistochemistry, quantitative real-time PCR, and western blot analysis. The effects of ER-phagy are investigated in HTR8/SVneo cells. Significantly increased levels of FAM134B, inositol-1,4,5-triphosphate receptor type 1 (IP3R), calnexin, cleaved caspase 3 and cytochrome C are detected in the PE placenta and sodium nitroprusside (SNP)-treated HTR-8/SVneo cells. Overexpression of FAM134B in HTR-8/SVneo cells results in increased apoptosis, impaired invasion capacity, and diminished mitochondrial function, while an autophagy inhibitor improves mitochondrial performance. Excessive ER-phagy is also associated with an increased concentration of gamma linolenic acid. Our findings suggest that FAM134B contributes to trophoblast apoptosis by mediating ER-mitochondria Ca ^2+^ transfer through mitochondria-associated endoplasmic reticulum membranes (MAMs) and subsequent mitochondrial function, further enhancing our understanding of PE etiology.

## Introduction

Preeclampsia (PE), a pregnancy-specific disease characterized by hypertension, proteinuria, and multiple organ dysfunction, affects up to 10% of pregnancies worldwide [1]. The primary cause of PE is placental malformation, which is resulted from vascular dysfunction and hypertrophic trophoblastic apoptosis [ 2– 10] . Therefore, maintaining normal trophoblast function plays a vital role in preventing PE.

It is recognized that shallow trophoblast invasion is associated with mitochondrial apoptosis [ 11, 12] . One type of mitochondrial apoptosis is induced by the entry of Ca ^2+^, which relies on the sustained increase in intracellular Ca ^2+^ [13]. The endoplasmic reticulum (ER) is where intracellular Ca ^2+^ is stored [14]. The physical communication site between the ER and mitochondria is defined as mitochondria-associated endoplasmic reticulum membranes (MAMs), which are conserved structures in eukaryotes. MAMs regulate the survival and death of cells by controlling Ca ^2+^ transfer via inositol-1,4,5-triphosphate receptor type 1 (IP3R) and the transport of other metabolites [15]. In recent years, studies have shown that MAMs are essential for autophagy by mediating the formation of autophagosomes, of which cytochrome C (Cyt C) is a biomarker [16]. Autophagy is imperative for maintaining homeostasis and plays a significant role in the onset and succession of PE [17]. Previous studies have revealed that FAM134B (family with sequence similarity 134, member B) is an ER receptor that binds to the autophagy modifiers LC3 and GABARAP, promoting ER-phagy. Overexpression of FAM134B triggers ER fragmentation, whereas downregulation of FAM134B expression in cells results in ER expansion [18]. In addition, FAM134B acts as a suppressor or promoter in different cancer types, such as esophageal cancer [19], colon cancer, and breast cancer [20]. The metabolic function of FAM134B in trophoblast cells has not been investigated despite its known role in tumor cell invasion, migration, and proliferation [21].

Furthermore, maternal lipid metabolism undergoes significant and rapid changes across gestation to fulfil the requirements for fetal development and growth [22]. Fatty acid induces apoptosis and oxidative stress and elevates the cytosolic level of oxidized mitochondrial DNA to activate the inflammasome [21]. However, the underlying mechanism mediated by FAM134B remains poorly understood.

In the present study, we aimed to investigate the role of FAM134B in placentas and HTR-8/SVneo cells, a cell line similar to human extravillous trophoblasts (EVTs). FAM134B expression, as well as its association with changes in fatty acids, was characterized, particularly with regard to its relationship with ER-phagy, Ca ^2+^ flux, and mitochondrial apoptosis.

## Materials and Methods

### Placental tissue collection

This study was conducted in accordance with the principles of the Declaration of Helsinki and was approved by the Research Ethics Committee of the First Affiliated Hospital of Chongqing Medical University, Chongqing, China (batch number: 2020-41). Informed consent was obtained from each participant. Pregnant women with PE ( *n*=10) and healthy pregnant women ( *n*=10) were included in this study. The definition of PE was consistent with the guidelines of the American College of Obstetrics and Gynecology [23]. Participants in the PE group met at least one of the following criteria: 1) systolic blood pressure ≥160 mmHg; 2) diastolic blood pressure ≥110 mmHg; and/or 3) proteinuria (based on a dipstick test of randomly collected urine specimens). Pregnant women were not eligible for this study if they had chronic hypertension, cardiovascular disease, gestational diabetes mellitus, chronic renal disease, collagen disorder, metabolic diseases, a history of smoking, or multiple pregnancies. Maternal characteristics such as age, body mass index (BMI) at delivery, gestational age, and parity were matched for the two groups. Fresh placental tissue was taken after caesarean sectioning. Five pieces were randomly taken from the maternal side of the placental villus tissue, avoiding the calcification region. The samples were immediately frozen in liquid nitrogen. After collection, the tissue on ice was transferred to the laboratory at the Department of Gynecology and Obstetrics in the First Affiliated Hospital of Chongqing Medical University.

### Cell culture, treatment, and transfection

The human-transformed primary extravillous trophoblast cell line (HTR8/SVneo) used in this study was kindly provided by Dr CH Graham (Queen’s University, Kingston, Canada). HTR8/SVneo cells were cultured in RPMI 1640 medium (C11875500BT; Life Technologies, New York, USA) supplemented with 10% fetal bovine serum (900-108; Gemini Bio-Products, West Sacramento, USA) and 1% penicillin-streptomycin at 37°C in 5% CO _2_. Sodium nitroprusside (SNP; Sigma, St Louis, USA) was made fresh and used at a final concentration of 2.5 mM for 6 h, as described in a previous study [24]. Stimulation of villus explants using SNP produces reactive oxygen species that simulate the pathophysiological state of PE [25]. Chloroquine (Sigma), an autophagy inhibitor, was used at a final concentration of 1 mM for 6 h. For siRNA transfection experiments, adherent cells were seeded in 12-well plates 18 to 24 h before lentivirus transfection at a concentration of 1×10 ^5^ cells/well. When the concentration of cells was approximately 2×10 ^5^ cells/well, siGENOME SMART pool siRNAs (Thermo Scientific, Waltham, USA) were incubated with the cells at a final concentration of 100 nM, according to the manufacturer’s instructions. After 24 h of incubtaion, the original medium was replaced by 2 mL of fresh medium containing 6 μL/mL polybrene. The cells were harvested 72 h after transfection when fluorescence was observed. The three groups were as follows: 1) HTR8/SVneo cells; 2) HTR8/SVneo ^FAM134B−^ cells whose *FAM134B* gene was knocked down; and 3) HTR8/SVneo ^FAM134B+^ cells overexpressing the *FAM134B* gene.

### Immunofluorescence staining

Cells were seeded onto coverslips 48 h before treatment and then permeabilized with blocking buffer [0.05% (w/v) saponin, 0.5% (w/v) BSA, 50 mM NH _4_Cl, and 0.02% NaN _3_ in PBS, pH 7.4] for 30 min, fixed in 4% paraformaldehyde for 30 min, and incubated with 5% BSA for 1 h. Then, the cells were incubated overnight at 4°C with the following primary antibodies: anti-FAM134B (1:1000 dilution; Abcam, Cambridge, UK), anti-cytochrome C (1:1000 dilution; Abcam), anti-IP3R (1:1000 dilution; Abcam), and washed three times with PBS. After incubation for 1 h with secondary antibodies (Alexa Fluor-labelled goat anti-rat A11077, goat anti-rabbit A11011/A11008, and goat anti-mouse A11001; Thermo Scientific), the cells were washed with PBS three times and then incubated for 30 min with 1 μM ER tracker (Invitrogen, Carlsbad, USA). The cells were subsequently mounted onto Vectashield (Vector Laboratories, Newark, USA) and treated with 4′,6-diamidino-2-phenylindole (DAPI). The slides were observed under a fluorescence microscope (EVOS FL Auto Imaging System; Life Technologies).

### Confocal microscopy

Scanning laser confocal microscopy was performed using a Leica TCS SP5 confocal microscope (Leica, Wetzlar, Germany) equipped with a Plan-Apochromat 63×/1.4 numerical aperture oil objective with a pixel size of 8.7 nm. Images were subjected to postacquisition Airyscan processing with ZenBlue software and colocalization analysis. Image presentation was performed using ImageJ software (National Institutes of Health, Bethesda, USA).

### Transmission electron microscopy (TEM)

The placental tissue was fixed with buffer containing 2.5% glutaraldehyde, 2.5% paraformaldehyde and 0.1 M sodium cacodylate (pH 7.4). The tissue was washed in 0.1 M cacodylate buffer and postfixed with 1% osmium tetroxide (OsO _4_)/1.5% potassium ferrocyanide (K _2_FeCN _6_) for 1 h. The tissue was then washed with water three times and incubated in 1% uranyl acetate in maleate buffer for 1 h, followed by three washes with maleate buffer and subsequent dehydration in increasing concentrations of ethanol (10 min each; 50%, 70%, 90%, and 100% twice). On the following day, the samples were embedded in TAAB 812 Resin mixture and polymerized at 60 °C for 48 h. The cells were then put into a buffer containing 1% glutaraldehyde and 0.2 M HEPES and fixed in uranyl acetate and OsO _4_. After dehydration in increasing ethanol concentrations (10 min each; 50%, 70%, 90%, and 100% twice), the sample was purged with propylene oxide, embedded in epoxy resin, and polymerized at 60°C for 72 h. Thin sections were cut from each sample with a Leica EMUC6 ultramicrotome (Leica), and images were taken using a transmission electron microscope (Titan; FEI, Hillsboro, USA).

### Western blot analysis

Proteins were extracted from frozen placental tissues and HTR8/SVneo cells with RIPA lysis buffer (Beyotime, Shanghai, China). Following the manufacturer’s protocol, the protein concentration was determined using a BCA Protein Assay kit (Beyotime). Western blot analysis was performed based on the technique established in our laboratory [26]. As previously described, protein samples were subjected to SDS polyacrylamide gel electrophoresis, resolved by electrophoresis, and transferred to polyvinylidene difluoride membranes (Millipore, Billerica, USA) [27]. The antibodies used were anti-FAM134B (1:1000 dilution; Abcam), anti-IP3R (1:1000 dilution; Abcam), anti-FACL4 (1:1000 dilution; Abcam), anti-cytochrome C (1:500 dilution; Abcam), anti-cleaved caspase-3 (1:1000 dilution; Abcam), anti-IP3R (1:1000 dilution; Abcam), anti-calnexin (1:1000 dilution; Abcam), anti-BNIP3 (1:1000 dilution; Abcam), anti-PSME2 (1:1000 dilution; Abcam), and anti-β-actin (1:1000 dilution; Abcam). All incubation was performed at 4°C overnight. The polyvinylidene difluoride membranes were then incubated with a secondary antibody conjugated with horseradish peroxidase (1:5000 dilution; Abcam). The protein bands were visualized using western bright ECL kit (Advansta, Menlo Park, USA) and quantified with the ChemiDoc™ XRS+(Bio-Rad, Hercules, USA).

### Quantitative real-time PCR

TsingZol (TaKaRa, Dalian, China) was used to isolate RNA. The RNA quality and quantity were assessed with a NanoDrop 2000 instrument (Thermo Scientific), and reverse transcribed into complementary DNA (cDNA) using high-capacity cDNA synthesis kit (TaKaRa) following the manufacturer’s instructions. PCR reactions were performed using the SYBR Green PCR kit (TaKaRa). The thermal cycling conditions were as follows: 50°C for 2 min, 95°C for 10 min, followed by 40 cycles of 94°C for 15 s and 60°C for 1 min. Gene expression was calculated using the 2 ^–∆∆Ct^ method [26], and the data were normalized to the expression of the reference gene glyceraldehyde 3-phosphate dehydrogenase ( *GAPDH*). The sequences of primers used are listed in Table 1.

**Table:** **Table 1** Sequences of primers used for qRT-PCR

Gene	Primer sequences (5′→3′)
*FAM134B*	Forward	GCAAAGAGACGCAATCAGCA
	Reverse	CTGCACACCCTCTAACTGG
*Cyto C*	Forward	TACTCTTACACAGCCGCCAA
	Reverse	TGAGATAAGCTATTAAGTCTGCC
*Caspase 3*	Forward	AATGACATCTCGGTCTGGTA
	Reverse	TCTTTAGAAACATCACGCATC
*FACL4*	Forward	CCGCTATCTCCTCAGACACA
	Reverse	CAACTCTGCCAGTAGTATAGTCA
*IP3R*	Forward	TGTTCTTTGTCAGCGATGTCC
	Reverse	CTCATCAGCTTCTGCCGTTC
*Calnexin*	Forward	GCCCTTCCTGTTTGACACC
	Reverse	GCAGTTTCACATAGGCACCAC
*PSME2*	Forward	CTTTTCCAGGAGGCTGAGGAAT
	Reverse	AGGGAAGTCAAGTCAGCCAC
*BNIP3*	Forward	UACUGCUGGACGCACAGCATT
	Reverse	UGCAGUGCGUCCAGCAGUATT
*FADS1*	Forward	CCGACATCATCCACTCACTAAA
	Reverse	AGTCTTCCTCCTCTTCTTCCA
*ELOVL5*	Forward	GCGTCCATACCTCTGGTGGAA
	Reverse	AATGTGCACGGCCAGATGA
*FADS2*	Forward	ACTTTGGCAATGGCTGGATTCCTACCCTC
	Reverse	ATCGGGATCCTTGTGGAAGATGTTAGG
*GAPDH*	Forward	CAAATTCCATGGCACCGTCA
	Reverse	GTTGTTGTAGCCAAATTCGTTGT

### Immunohistochemistry analysis

Immunohistochemistry (IHC) analysis was performed as previously described [28]. In brief, 5-μm paraffin-embedded tissue sections were deparaffinized in xylene, rehydrated in a serial gradient of ethanol, and washed with PBS. After the endogenous peroxidase activity was quenched sequentially using 3% hydrogen peroxide for 15 min at room temperature, the antigens in the tissue sections were further retrieved with citrate buffer. The slides were then incubated at 4°C overnight with monoclonal mouse anti-FAM134B (1:50 dilution; Abcam) or polyclonal rabbit anti-FAM134B (1:50 dilution; Abcam) antibodies. After reheating to room temperature for 30 min, the slides were rinsed with PBS and then incubated separately with polymer helper and polyperoxidase-anti-mouse/rabbit IgG for 20 min at 37°C. 3,3′-Diaminobenzidine (ZSGB-BIO, Beijing, China) was used as the chromogen, and hematoxylin (Beyotime) was used for nuclear counterstaining. Primary antibodies were omitted for negative control samples. Each experiment was repeated three times.

### Transwell assay

The invasion capacity of the cells was evaluated using a Matrigel invasion chamber. First, cell inserts (Costar; Corning Co., Corning, USA) were coated with 100 μL of basement membrane matrix (1:8 dilution; BD Biosciences, Franklin Lakes, USA) and placed in a 24-well plate at 4°C overnight. The chamber for the migration test was not pre-coated with Matrigel. Invasion experiments were performed using 6×10 ^5^ HTR8/SVneo cells. A total of 150 μL of cell suspension was placed in the upper chamber, and 750 μL of medium containing 10% fetal bovine serum was placed in the lower chamber. After 24 h of culture, the cells on the apical side were removed with cotton swabs, while cells on the basal side were rinsed with PBS, fixed in methanol, and subjected to crystal violet staining (Beyotime). Finally, the invaded or migrated cells were observed using an optical microscope (Life Technologies) and the number of cells was counted. The experiment was repeated three times for each group.

### Measurement of cytosolic Ca ^2+^

Cytosolic Ca ^2+^ levels were determined using Fluo-3 AM (Invitrogen). In brief, cells were treated with 5 M Fluo-3 AM in Hank’s balanced salt solution (HBSS) for 60 min at 37°C. Fluorescence images were obtained using an Olympus IX70 microscope with 40× objectives (Olympus, Tokyo, Japan) and alternately illuminated with 340 and 380 nm light for 250 ms (Lambda DG-4; Sutter Instrument Co., Novato, USA). Light with emission wavelengths >510 nm was captured using a CCD camera (Orca-ER; Hamamatsu Photonics, Shizuoka, Japan). Images were collected every 5 s. Background correction and image analysis were performed with SlideBook (Intelligent Imaging Innovations, Denver, USA).

### Reactive oxygen species (ROS) production assay

The cells were washed and incubated with 3 μM of superoxide indicator (Mitosox; Thermo Scientific) in a medium containing: 121 mM of NaCl, 10 mM of NaHCO _3_ and HEPES, 4.7 mM of KCl, 10 mM of KH _2_PO _4_, MgSO _4_, CaCl _2_ and glucose, and 2% dextran at 37°C for 30 min. After incubation, cells were washed once with 2 mL of warm HBSS buffer and centrifuged (5 min, 500 *g*) to remove any excess Mitosox. Then cells were resuspended in HBSS using an appropriate volume to obtain a single-cell suspension and analyzed by flow cytometery. In flow cytometry analysis, an excitation wavelength of 510 nm and an emission wavelength of 580 nm were used to detect oxidized mitosox reagent on a FACSCalibur ^TM^ flow cytometer (BD Biosciences). The signals were quantified with ImageJ software (National Institutes of Health).

### Mitochondrial oxygen consumption assay

Real time mitochondrial oxygen consumption rate (OCR) was measured using the XF24 extracellular flux analyzer (Seahorse Biosciences, North Billerica, USA). Cells were seeded on collagen-coated XF24 plates (Seahorse Bioscience) at 4×10 ^4^ cells/well. Cells were kept overnight in normal growth medium and then rinsed twice and kept in 600 μL of sodium bicarbonate-free DMEM medium (Sigma) supplemented with 20 mM glucose and 1 mM sodium pyruvate. The respiratory rate was measured at 37°C in 8 replicates (independent wells) for each of the plates. OCR was calculated and analyzed using the Seahorse XF24 v1.7.0.74 software.

### Flow cytometry analysis

After treatment, the cells (1×10 ^5^ cells/mL) were collected in 15 mL tubes and stained with Annexin V-APC and propidium iodide (PI) reagents (KeyGen BioTech, Nanjing, China) for 15 min at room temperature in the dark. The degree of apoptosis in the three cell groups was then rapidly analyzed using an FSCAN flow cytometer (BD Biosciences).

### RNA sequencing

Total RNA was extracted from HTR8/SVneo cells and treated cells, and mRNA was enriched using magnetic beads with oligo(dT). Random hexamers were used to synthesize the first cDNA strand, and the second cDNA strand was synthesized by adding buffer, dNTPs, RNase H, and DNA polymerase I. A QiaQuick PCR kit (Qiagen, Dusseldorf, Germany) was used to purify the library, EB buffer was added for elution, and end repair was performed. Base A was added, and a sequence splice was added. Agarose gel electrophoresis was performed to recover the target fragment, and PCR was performed to complete the library preparation. The constructed library was sequenced on an Illumina HiSeq 3000 platform (Illumina, Shanghai, China).

### Chemical derivatization and gas chromatography-mass spectrometry (GC-MS) analysis

Metabolites were extracted from HTR8/SVneo cells, HTR8/SVneo ^FAM134B−^ cells, and HTR8/SVneo ^FAM134B+^ cells using 2 mL of methanol/toluene (4:1 v/v) solution containing nonadecanoic acid (20 μg/mL; Nu-Chek Prep, Inc., Elysian, USA) and tridecanoic acid (20 μg/mL; Nu-Chek Prep, Inc.) as internal standards (ISs). The extract was evaporated to dryness with a rotary vacuum dryer. The dried specimens were chemically derivatized using the methyl chloroformate (MCF) approach [29]. The metabolites were identified by comparing their mass spectra and retention time against that of an in-house MCF library and lipid mass spectra libraries. The MCF derivatives were analyzed in an Agilent GC7890B system (Agilent, Santa Clara, USA) coupled to an MSD5977A mass selective detector (EI) set at 70 eV. The GC column used for metabolite analysis was a ZB-1701 GC capillary column (30 m× 250 μm id× 0.15 μm with 5 m guard column; Phenomenex, Inc., Torrance, USA). The GC analysis parameters were set as described in Smart *et al*. [30]. GC-MS chromatographic peaks were deconvoluted via an automated mass spectral deconvolution and identification system (AMDIS). The relative quantification of identified metabolites was performed using an XCMS-based R script by selecting the most abundant reference ion within a correct retention time bin. The metabolite levels were normalized using D _4_-alanine, and the biomass was used to correct the dilution effect.

### ELISA

Cell supernatant samples were collected for detection. Phosphate-buffered saline (PBS) was added to the cells at various proportions. Following centrifugation, the supernatant was collected for further analysis. The standardized operation was carried out according to the instructions from the following kits: human gamma-linolenic acid ELISA kit (Cat. F112070-B; FANKEW, Shanghai, China), human Δ6 desaturase (Cat. F112071-B; FANKEW), human Δ5 desaturase (Cat. F112072-B; FANKEW), human PGI2 ELISA kit (Cat. E-EL-0022c; Elabscience Biotechnology, Wuhan, China), and human TXA2 ELISA kit (Cat. E-EL-0057c; Elabscience Biotechnology).

### Statistical analysis

Statistical analyses were performed using GraphPad Prism (GraphPad Software, San Diego, USA). Data were analyzed using unpaired Student’s *t* test or one-way ANOVA (analysis of variance) followed by post hoc tests. Data are expressed as the mean±standard error of the mean (SEM) and. *P*<0.05 was considered statistically significant. Principal component analysis (PCA) was performed on the metabolomic data using the mixOmics R-package.

## Results

### Increased expression of FAM134B in PE placentas

**Figure 1:**
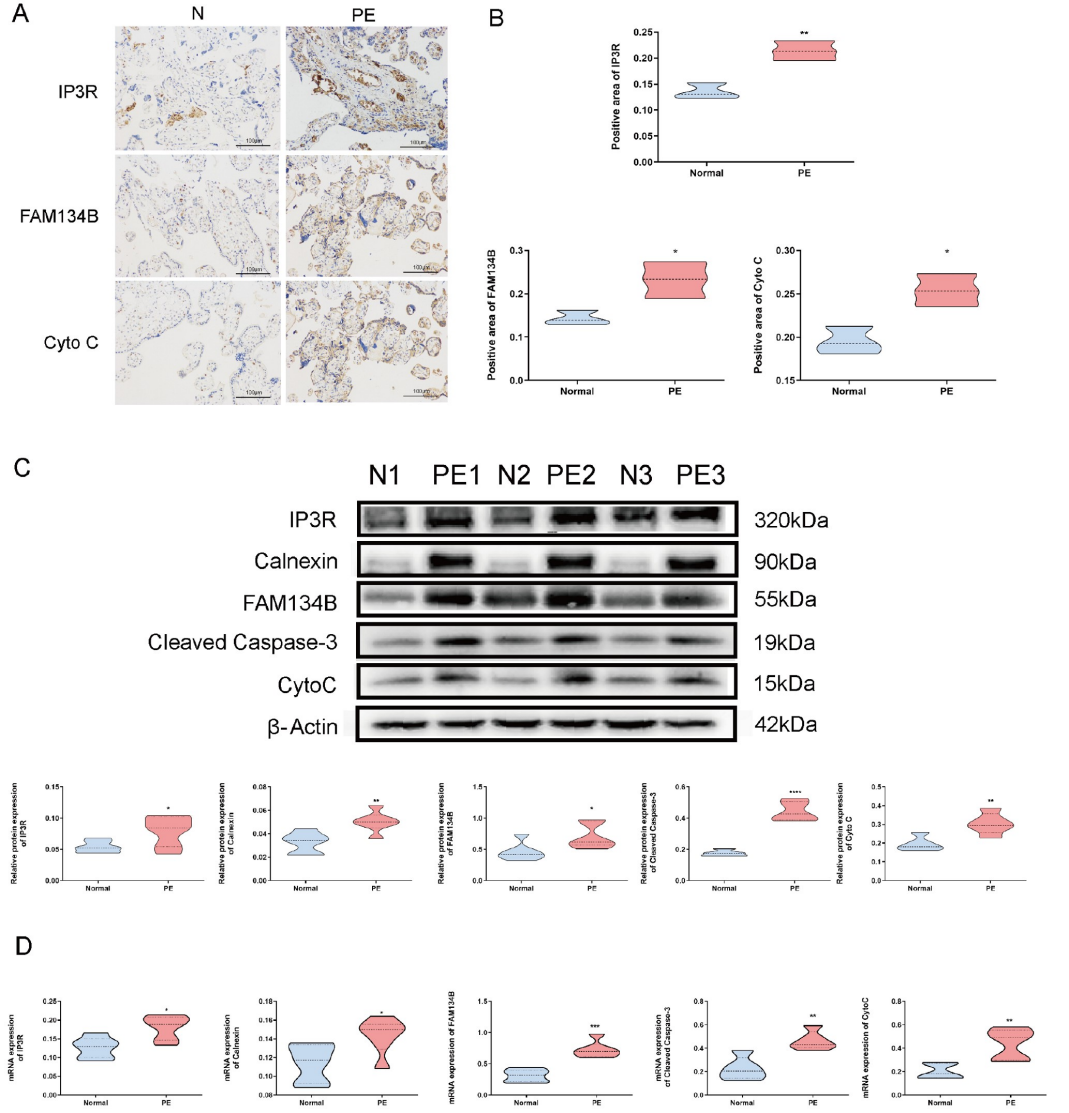
Figure 1 ER-phagy, Ca ^2+^ channels, and mitochondrial apoptosis in placental tissues (A) Immunohistochemistry (200×) of FAM134B, IP3R and Cyto C showing the levels in the placentas of normal pregnant women and pregnant women with PE. (B) Statistical analysis of the immunohistochemistry results. (C) Western blot analysis of FAM134B, IP3R, calnexin, cleaved caspase 3 and Cyto C in placental tissues from normal pregnant women and pregnant women with PE. (D) The statistical results of qRT-PCR. Data are presented as the mean±SEM of three independent experiments (unpaired Student’s t test; * P<0.05, ** P<0.01, *** P<0.001, **** P<0.0001).

The expressions of FAM134B, IP3R, and Cyto C were greater in the placental tissues of women with PE than in those of women with a normal pregnancy, as determined by immunohistochemistry ( Figure 1A,B). The function of MAMs was assessed by detecting the presence of specific markers: FAM134B for the ER, calnexin, and IP3R for MAMs and Cyto C and cleaved caspase 3 for mitochondria. Western blot analysis revealed increases in the expressions of FAM134B, IP3R, calnexin, cleaved caspase 3, and Cyto C in the placentas of PE patients ( Figure 1C), which was also confirmed by qRT-PCR ( Figure 1D).

### Oxidative stress increases ER-phagy and mitochondrial apoptosis via Ca ^2+^ channels in HTR8/SVneo cells

**Figure 2:**
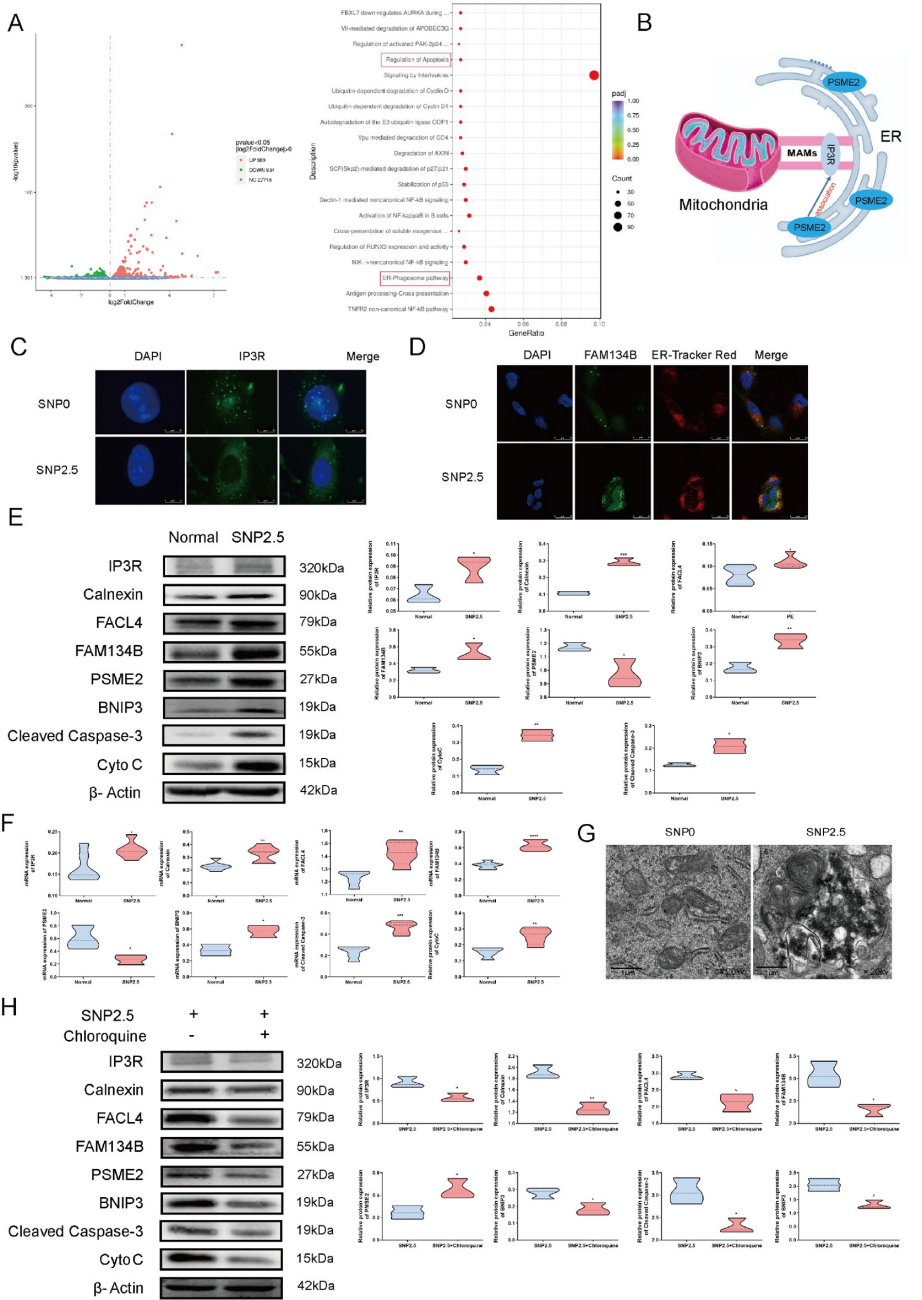
Figure 2 Oxidative stress increases ER-phagy, Ca ^2+^ channels, and mitochondrial apoptosis in HTR8/SVneo cells (A) Overall changes in genes between the SNP treatment group and the normal group. Red represents high expression, blue indicates low expression, and the Reactome dot of the pathway between the SNP treatment group and the normal group. (B) The role of PSME2 in the ER and MAMs. (C) Localization and levels of IP3Rs (green) and nuclei (blue). (D) Localization and density of FAM134B (green), the ER (red) and nuclei (blue). (E) Western blot analysis of FAM134B, IP3R, calnexin, FACL4, cleaved caspase 3 and Cyto C in HTR8/SVneo cells with or without SNP treatment. (F) The statistical results of qRT-PCR. (G) Representative transmission electron microscopy (TEM) images of the two groups. (H) Western blot analysis of HTR8/SVneo cells treated with SNP and SNP+chloroquine. Reactome analyses of DEGs after SNP treatment. Padj, corrected P value; Count, gene count. Data are presented as the mean±SEM of three independent experiments (unpaired Student’s t test; * P<0.05, ** P<0.01, *** P<0.001, **** P<0.0001).

To explore the molecular mechanism of ER-phagy, we performed RNA sequencing and identified 1923 DEGs in HTR8/SVneo cells subjected to SNP treatment, including 989 upregulated genes and 934 downregulated genes. Reactome analysis indicated that 41 genes are associated with the ER-phagosome pathway, and 30 are linked to apoptosis regulation. Gene set enrichment analysis confirmed the upregulation of both the ER-phagosome pathway and apoptosis regulation following SNP treatment ( Figure 2A). The differential gene expression results of the ER-phagosome pathway and regulation of apoptosis are displayed in Supplementary Figure S1A,B. Intriguingly, proteasome-related gene expression was significantly elevated in cells treated with SNP compared to that in normal cells. Specifically, the expression of proteasome activator subunit 2 (PSME2) was augmented in both the ER-phagosome pathway and the apoptosis pathway. The results indicated that PSME2 is a key gene in SNP treatment. As shown in Figure 2B, previous studies have shown that PSME2 is present in ER membrane fractions and interacts with the integral membrane substrate IP3R [31]. In addition, increased expression of PSME2 is associated with cancer invasion through the regulation of autophagy [32]. In summary, PSME2 is positively correlated with the occurrence and development of the ER-phagosome pathway and apoptosis pathway in mitochondria via MAMs, indicating that *PSME2* may be the intermediate gene that links MAMS to the ER. Furthermore, we performed a KEGG pathway analysis utilizing our RNA-seq data, which revealed that oxidative stress induced the degradation and elongation of fatty acids ( Supplementary Figure S2A,B), suggesting the potential involvement of fatty acid metabolism in this mechanism.

Immunofluorescence data revealed increased expression of IP3R in HTR8/SVneo cells treated with 2.5 mM SNP, which produces reactive oxygen species that simulate the pathophysiological state of PE, compared with that in untreated cells ( Figure 2C). Independent evaluation using immunofluorescence staining revealed that FAM134B levels were significantly increased in HTR8/SVneo cells exposed to SNP ( Figure 2D). Western blot analysis and qRT-PCR results revealed dramatic increases in the levels of FAM134B and PSME2 in ER-pregnant MAMs, IP3R, calnexin, and FACL4 in MAMs, and cleaved caspase 3 and Cyto C during mitochondrial apoptosis in response to oxidative stress ( Figure 2F). Next, we used TEM to investigate whether oxidative stress causes ER-phagy in HTR8/SVneo cells. As illustrated in Figure 2G, SNP treatment was associated with the appearance of ER-phagy in HTR8/SVneo cells. Treatment of HTR8/SVneo cells with the autophagy inhibitor chloroquine and oxidative stress markedly decreased the protein levels of FAM134B and PSME2 during ER-phagy; the protein levels of IP3R, calnexin and FACL4 in MAMs; and the protein levels of cleaved caspase 3 and Cyto C during mitochondrial apoptosis ( Figure 2H).

### Overexpression of FAM134B affects the formation of MAMs and Ca ^2+^ transfer

**Figure 3:**
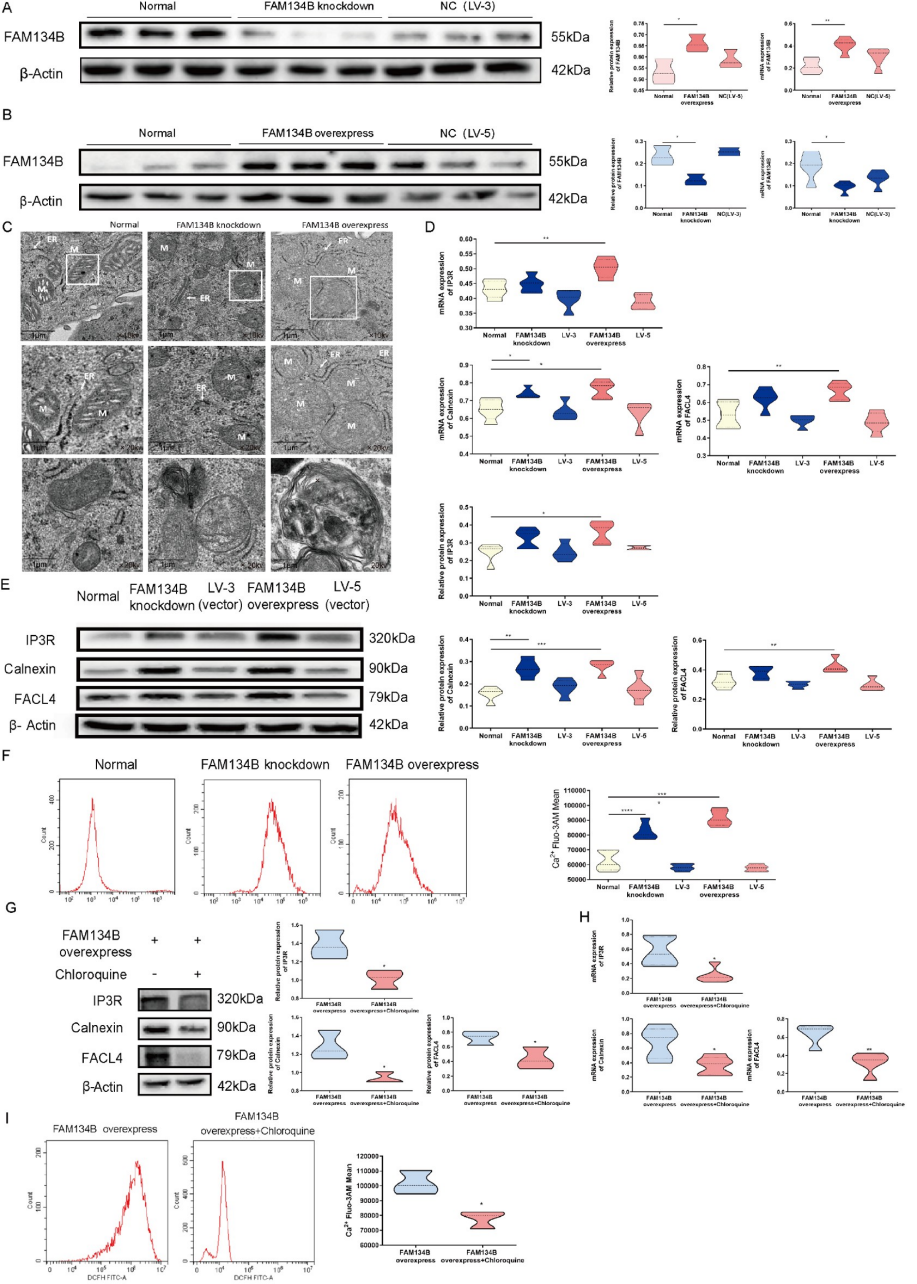
Figure 3 Aberrant ER-phagy influences MAM formation and Ca ^2+^ transfer in HTR8/SVneo cells (A) FAM134B knockdown in HTR8/SVneo cells following siRNA transfection, as determined by western blot analysis and qRT-PCR. (B) FAM134B overexpression in HTR8/SVneo cells following lentiviral infection, as determined by western blot analysis and qRT-PCR. (C) Representative transmission electron microscopy (TEM) images of the three groups at 6800 nm; ER: endoplasmic reticulum, M: mitochondria. (D) The statistical results of qRT-PCR analysis of IP3R, calnexin and FACL4 expression. (E) Western blot analysis of IP3R, calnexin and FACL4. (F) Flow cytometry analysis of Ca 2+ Fluo-3 AM in HTR8/SVneo cells following siRNA transfection. (G) Western blot analysis of HTR8/SVneo FAM134B+ cells following treatment with chloroquine. (H) The statistical results of qRT-PCR analysis of HTR8/SVneo FAM134B+ cells following treatment with chloroquine. (I) Flow cytometry analysis of Ca 2+ Fluo-3 AM in HTR8/SVneo FAM134B+ cells following treatment with chloroquine. Data are presented as the mean±SEM of three independent experiments (one-way ANOVA with post hoc test; * P<0.05, ** P<0.01, *** P<0.001, **** P<0.0001).

To characterize the mechanism underlying ER-phagy in MAMs and mitochondrial apoptosis, we knocked down and overexpressed the *FAM134B* gene in HTR8/SVneo cells (HTR8/SVneo ^FAM134B−^ and HTR8/SVneo ^FAM134B+^ groups). We showed a notable reduction of FAM134B in HTR8/SVneo ^FAM134B−^ cells ( Figure 3A) and an increase of FAM134B in HTR8/SVneo ^FAM134B+^ cells, as measured by western blot analysis and qRT-PCR ( Figure 3B). To investigate the morphology of the ER and mitochondria and their physical interactions during ER-phagy, TEM was utilized to examine the HTR8/SVneo, HTR8/SVneo ^FAM134B−^ and HTR8/SVneo ^FAM134B+^ cells. As shown in Figure 3C, obvious ER autophagosomes were found in the HTR8/SVneo ^FAM134B+^ cells. Furthermore, the ER membrane displayed a disorganized morphology in cells affected by excessive ER-phagy, and a marked increase in the proportion of mitochondria in the ER was detected in the HTR8/SVneo ^FAM134B+^ cells. ER expansion and mitochondrial swelling were detected in the HTR8/SVneo ^FAM134B-^ cells.

In addition, elevated mRNA and protein expressions of IP3R, calnexin, and FACL4 were detected in the HTR8/SVneo ^FAM134B+^ group ( Figure 3D,E). In addition, we observed that compared to those in the control group, the levels of cytoplasmic Ca ^2+^ in the HTR8/SVneo ^FAM134B−^ cells and especially in the HTR8/SVneo ^FAM134B+^ cells were significantly increased in the ER-phagy group ( Figure 3F). Given that chloroquine inhibits autophagy, we speculated that chloroquine might protect against FAM134B overexpression-mediated aberrant Ca ^2+^ via MAMs. Chloroquine strongly inhibited the expression of Ca ^2+^ channels ( Figure 3G,H) and suppressed cytoplasmic Ca ^2+^ in HTR8/SVneo ^FAM134B+^ cells ( Figure 3I). Collectively, these results indicated that excessive ER-phagy promoted excessive Ca ^2+^ release.

### FAM134B overexpression dramatically induces mitochondrial apoptosis

**Figure 4:**
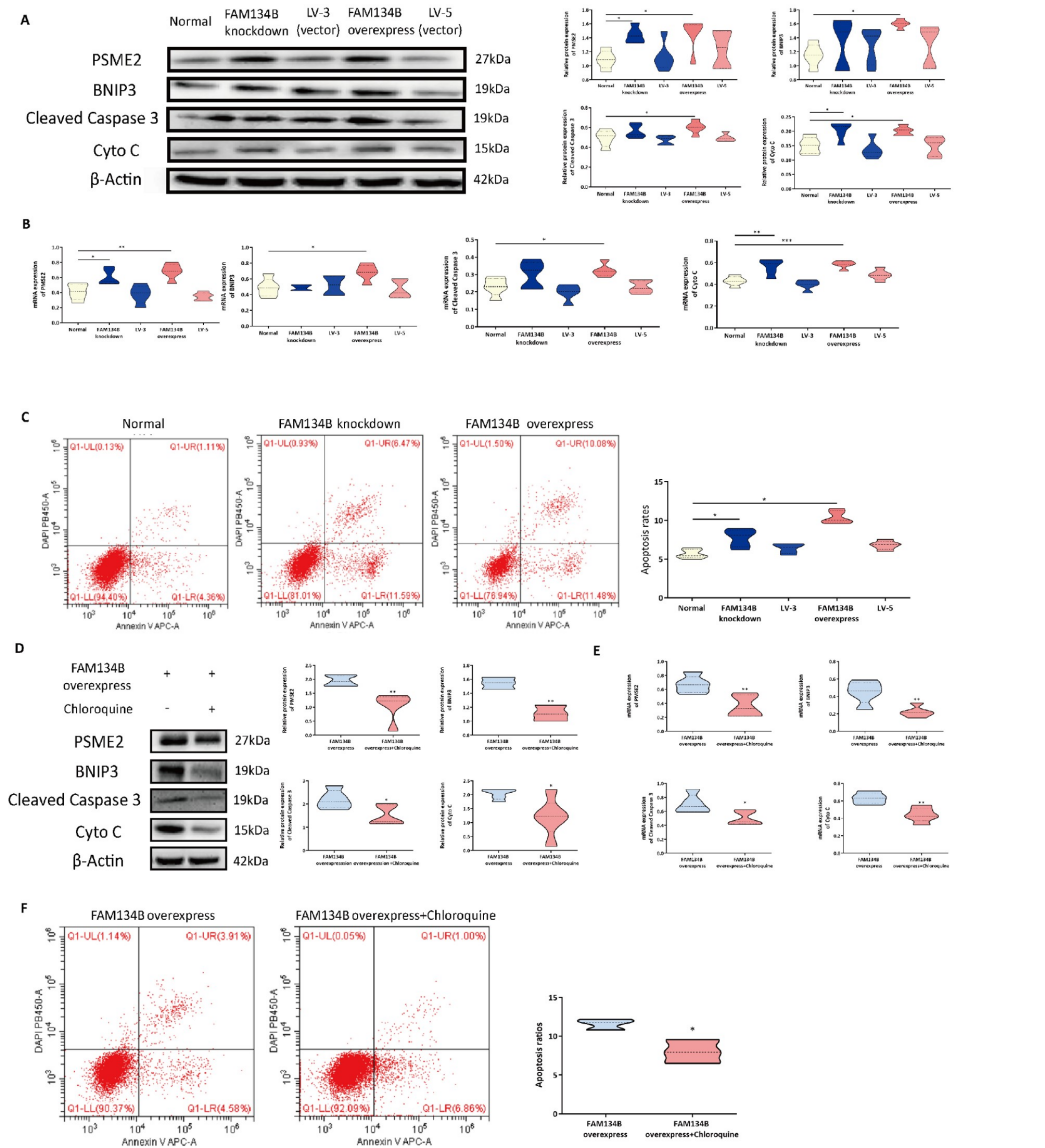
Figure 4 FAM134B overexpression promotes the apoptosis of HTR8/SVneo cells (A) PSME2, BNIP3, cleaved caspase 3 and Cyto C expressions were determined by western blot analysis in HTR8 cells following transfection with siRNAs. The statistical results of qRT-PCR analysis. (C) Flow cytometry analysis of apoptosis in HTR8/SVneo cells following siRNA transfection. (D) Western blot analysis of HTR8/SVneo FAM134B+ cells following treatment with chloroquine. (E) The statistical results of qRT-PCR analysis of HTR8/SVneo FAM134B+ cells following treatment with chloroquine. (F) Flow cytometry analysis of apoptosis in HTR8/SVneo FAM134B+ cells following treatment with chloroquine. Data are presented as the mean±SEM of three independent experiments (one-way ANOVA with post hoc test, * P<0.05, ** P<0.01).

Next, to assess whether ER-phagy is also responsible for activating mitochondrial apoptosis, we measured the levels of key mediators, including BNIP3, PSME2, cleaved caspase 3, and Cyto C, in the HTR8/SVneo ^FAM134B−^ and HTR8/SVneo ^FAM134B+^ groups. The western blot analysis and qRT-PCR results demonstrated that the expressions of BNIP3, PSME2, cleaved caspase 3, and Cyto C were greater in the HTR8/SVneo ^FAM134B+^ cells than in the control cells ( Figure 4A,B). Excessive FAM134B expression significantly induced mitochondrial apoptosis. The apoptosis ratio in the three groups determined by flow cytometry showed consistent trends, namely, more apoptosis occurred in the HTR8/SVneo ^FAM134B−^ and HTR8/SVneo ^FAM134B+^ cells than in the normal cells ( Figure 4C). Chloroquine markedly suppressed mitochondrial apoptosis in HTR8/SVneo ^FAM134B+^ cells ( Figure 4D–F). These results suggest that excessive ER-phagy can aggravate mitochondrial apoptosis in HTR8/SVneo cells.

### Overexpression of FAM134B weakens mitochondrial function

**Figure 5:**
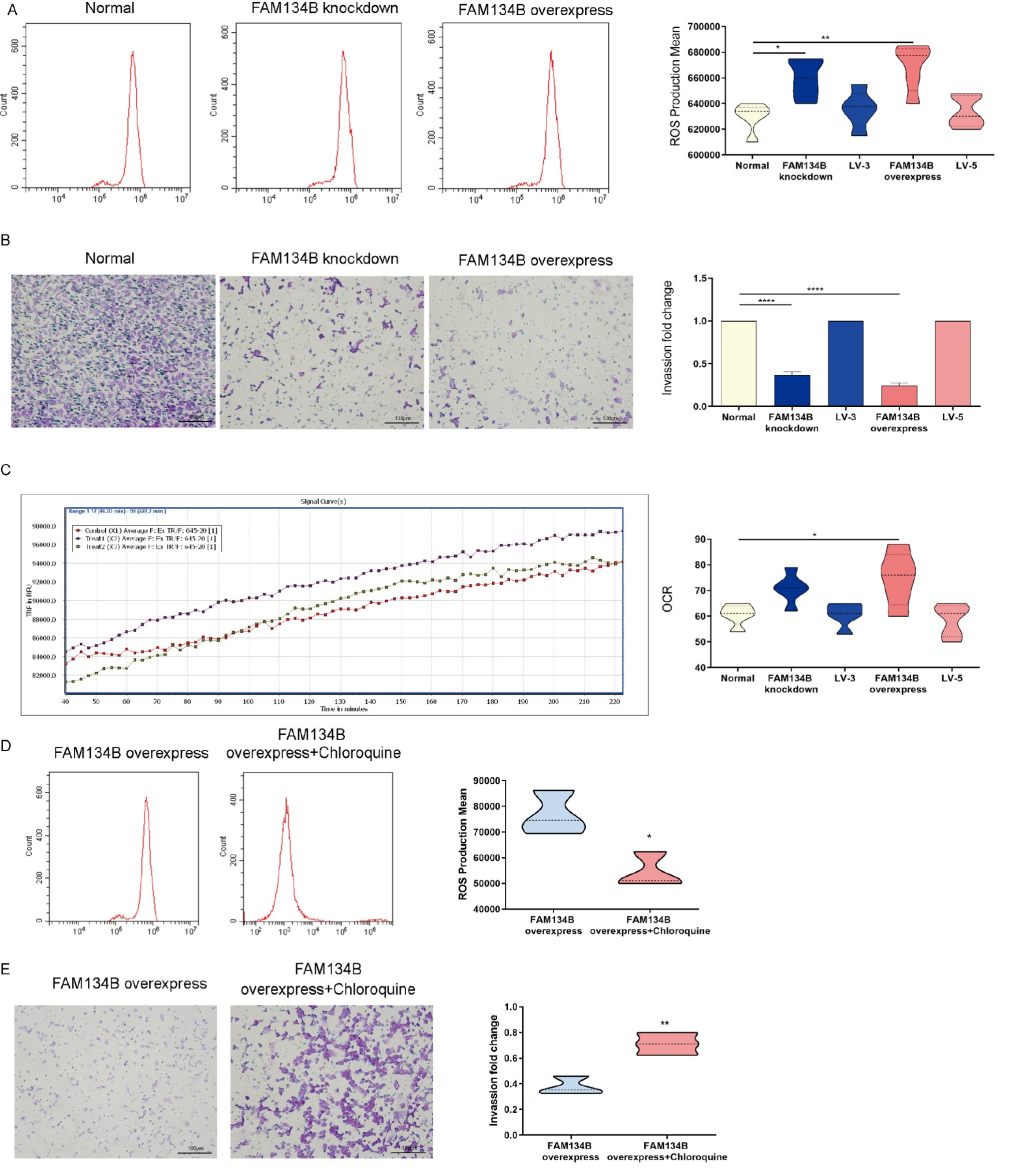
Figure 5 FAM134B overexpression weakens the mitochondrial function of HTR8/SVneo cells (A) Flow cytometry analysis of ROS production in HTR8/SVneo cells following siRNA transfection. (B) The invasion capacity of HTR8/SVneo cells following siRNA transfection. (C) Mitochondrial oxidative capacity of HTR8/SVneo cells following siRNA transfection. (D) Flow cytometry analysis of ROS production in HTR8/SVneo FAM134B+ cells following treatment with chloroquine. (E) Mitochondrial oxidative capacity of HTR8/SVneo FAM134B+ cells following treatment with chloroquine. Data are presented as the mean±SEM of three independent experiments (one-way ANOVA with post hoc test; * P<0.05, ** P<0.01, **** P<0.0001).

ROS generation was significantly greater in the HTR8/SVneo ^FAM134B+^ group than in the control group ( Figure 5A). In addition, the invasion capacity of both HTR8/SVneo ^FAM134B−^ and HTR8/SVneo ^FAM134B+^ cells decreased markedly compared with that of normal HTR8 cells according to the Matrigel cell invasion assay ( *P*<0.001); moreover, the invasion ability of HTR8/SVneo ^FAM134B+^ cells was lower than that of HTR8/SVneo ^FAM134B−^ cells ( Figure 5B). Compared with HTR8/SVneo cells, HTR8/SVneo ^FAM134B+^ cells displayed a modest but significant improvement in mitochondrial oxidative capacity ( Figure 5C). We also treated HTR8/SVneo ^FAM134B+^ cells with chloroquine and found that ROS production was increased ( Figure 5D) and that chloroquine promoted invasion in HTR8/SVneo ^FAM134B+^ cells ( Figure 5E). These data suggested that aberrant ER-phagy weakens mitochondrial function in trophoblast cells.

### Overexpression of FAM134B affects fatty acid metabolism.

**Figure 6:**
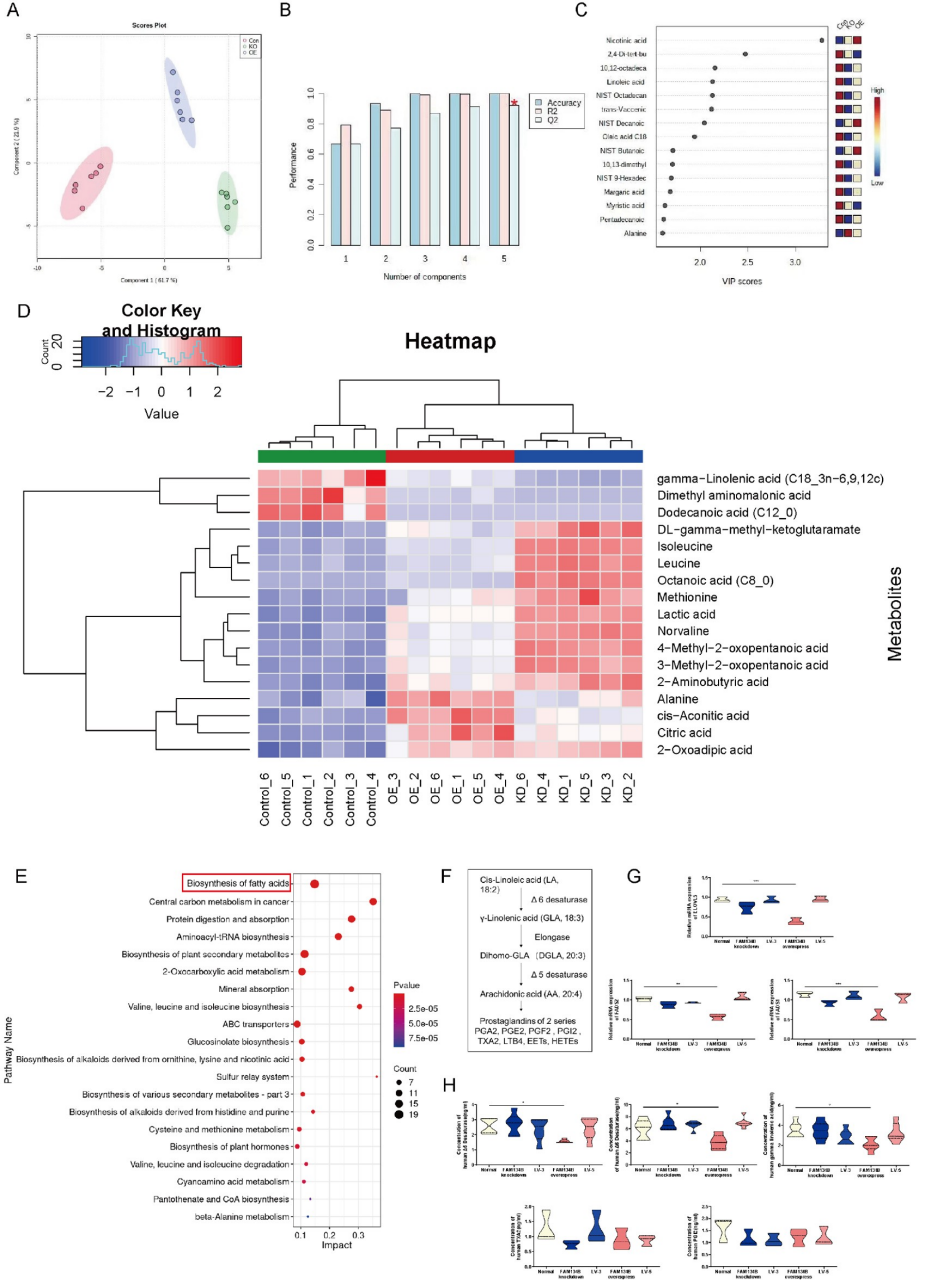
Figure 6 Metabolomic analysis and functional experiments of the three cell groups (A) PCA of the three groups showing a clustering pattern. (B) Validation parameters of the analysis. (C) Metabolites were significantly different among the three groups. (D) Concentrations of identified metabolites in the groups. (E) Enrichment analyses of metabolic profiles among the three groups. (F) Metabolism of essential fatty acids. (G) The statistical results of qRT-PCR. (H) Statistical analysis of the ELISA results. The relative concentration of metabolites was illustrated using a log 2 scale. Fold changes in the concentrations of metabolites related to HTR8/SVneo controls are shown in red (increased levels in the HTR8/SVneo FAM134B- and HTR8/SVneo FAM134B+groups) and blue (decreased levels in the HTR8/SVneo FAM134B- and HTR8/SVneo FAM134B+groups). Only the metabolites with P<0.05 (Tukey’s HSD), q<0.05 (false discovery rates) and VIP>1 are shown. KEGG pathway analyses of DEGs after FAM134B treatment. P  value, probability value; Count, metabolite counts. Data are presented as the mean±SEM of three independent experiments (one-way ANOVA with post hoc test, * P<0.05, ** P<0.01, *** P<0.001).

To investigate the relationship between fatty acid profiles and excessive ER-phagy, we detected a total of 139 GC-MS peaks in three cell groups (HTR8/SVneo, HTR8/SVneo ^FAM134B−^ and HTR8/SVneo ^FAM134B+^), 92 of which were identified using the Villas-Bôas MCF MS library. Principal component analysis (PCA) revealed that the HTR8/SVneo, HTR8/SVneo ^FAM134B−^ and HTR8/SVneo ^FAM134B+^ cells clustered separately; principal component (PC)1 and PC2 explained 61.7% and 21.9% of the variance, respectively ( Figure 6A); and the validation parameters of the six PCs were as follows: R2=0.96 and Q2=0.77 ( Figure 6B). Univariate analysis revealed that 15 metabolites were significantly different between the HTR8/SVneo, HTR8/SVneo ^FAM134B−^ and HTR8/SVneo ^FAM134B+^ groups ( *P* values and q values<0.05; Figure 6C). The majority of the metabolites were detected at lower concentrations in samples from HTR8/SVneo ^FAM134B+^ cells than in those from HTR8/SVneo ^FAM134B-^ cells. The six metabolites detected at significantly lower concentrations in the HTR8/SVneo ^FAM134B+^ cells were γ-linolenic acid (GLA), dimethyl aminomalonic acid, dodecanoic acid, isoleucine, leucine, and octanoic acid. In contrast, four metabolites were detected at significantly higher concentrations in the HTR8/SVneo ^FAM134B+^ cells: alanine, cis-aconitic acid, citric acid, and 2-oxoadipic acid ( Figure 6D). Next, we performed KEGG enrichment analyses among the three groups of cells to identify the pathways and cell functions associated with these continuously altered genes. As shown in Figure 6E, 92 GC-MS peaks were identified that were primarily enriched in various metabolic pathways, including fatty acid biosynthesis, central carbon metabolism in cancer, and protein digestion and absorption. GLA plays a crucial role in fatty acid metabolism. Linolenic acid (LA) is converted to GLA (18:3, n-6) by the enzyme Δ6 desaturase, and GLA is elongated to form dihomo-GLA (DGLA, 20:3, n-6), the precursor of the 1 series of PGs ( Figure 6F). The expressions of the fatty acid metabolism-related enzymes ELOVL5, FADS1 and FADS2 were significantly decreased in the HTR8/SVneo ^FAM134B+^ cells ( Figure 6G). Similarly, FAM134B overexpression prominently reduced the concentrations of Δ5 desaturase, Δ6 desaturase, and GLA, as detected by ELISA, but did not alter the concentrations of TXA2 or PGI2 ( Figure 6H).

## Discussion

In this study, we demonstrated the mechanism by which FAM134B-mediated ER-phagy contributes to elevated calcium flux from the ER destined for mitochondria, which in turn induces apoptosis. We found that MAMs, by interacting with the ER and mitochondria, transport increased Ca ^2+^ released from excessive ER-phagy, resulting in impaired mitochondrial function. The latter may interfere with placentation, causing placental ischemia and, subsequently, PE.

Autophagy is indispensable for maintaining cell and tissue homeostasis and counteracts both the onset and progression of many disease conditions [ 33, 34] . In some situations, autophagic activity might be defective and may not successfully eliminate impaired cellular components. In other circumstances, autophagy may be overwhelmed, resulting in ‘autophagic cell death’. Previous reports have suggested that impaired autophagy in placental beds is involved in the pathophysiology of PE [35]. The sequestration of cytoplasmic material occurs via engulfment into endosomes or lysosomes in microautophagy [ 36, 37] . ER-phagy transports cytoplasmic components into the lysosome for degradation, which is a critical ER remodeling process [38]. Notably, FAM134B was identified as an ER-phagy receptor that mediates the turnover of portions of the ER via autophagy, and overexpression of FAM134B leads to ER fragmentation and lysosomal degradation [18]. In this study, we revealed increased expression of the ER-residing autophagy receptor FAM134B in the PE placenta and that oxidative stress increased the FAM134B level in HTR8/SVneo cells. HTR8/SVneo cells, a model of human extravillous trophoblast cells, are extensively used to evaluate the proliferation and migration processes of extravillous trophoblast cells [39] and to investigate the factors involved in PE [40]. These findings indicate that excessive ER-phagy mediated by FAM134B may be associated with the pathogenesis of PE.

In addition, the ER plays an essential role as a dynamic intracellular Ca ^2+^ reservoir that controls cytosolic calcium levels [14]. Ca ^2+^ release-activated Ca ^2+^ channels are activated by Ca ^2+^ released from the ER by channels formed by IP3Rs. We have shown elevated levels of calcium released in the PE placenta. One of the frequently investigated mechanisms of autophagy induction, involving the modulation of calcium channel activity, is IP3R [41]. IP3R1 is a Ca ^2+^ channel protein that controls the release of Ca ^2+^ from the ER [42]. We found that the expression of IP3R increased with the occurrence of ER-phagy in the oxidative stress cell model, suggesting that an increase in Ca ^2+^ flow occurs during ER-phagy. Ca ^2+^ released into the cytoplasm through IP3Rs was reported to play a role in both the inhibition and activation of autophagy, which is likely dependent on the cellular state [43]. Furthermore, we found that FAM134B overexpression increased the cytosolic Ca ^2+^ concentration, whereas the autophagy inhibitor decreased the Ca ^2+^ concentration in HTR8/SVneo ^FAM134B+^ cells. These results suggest that FAM134B is a key mediator of ER-phagy and ER-phagy results in excessive Ca ^2+^ release from the ER.

There is evidence that the disruption of calcium homeostasis can evoke different types of cell death [44]. We have provided biochemical evidence indicating that MAMs play an important role in the interaction between ER-phagy and cell death in the pathogenesis of PE. This interaction seems to occur within the ER membrane, since it is regulated by the transmembrane regions of the RHD of FAM134B. MAMs are thought to be critically important for mitochondrial metabolism, as Ca ^2+^ is directly transferred from the ER storage area to the mitochondria via these sites [45]. The regulation of MAMs impacts not only calcium flux but also mitochondrial function [ 46– 49] . The effect of Ca ^2+^ regulation on mitochondrial function is likely resulted from the engagement of MAMs. Our TEM results revealed a disorganized morphology of the ER and swollen mitochondria in FAM134B-overexpressing cells. We observed a marked increase in ER apposition to mitochondria, suggesting that FAM134B is critical for the conformational changes of MAMs. Our analysis of HTR8/SVneo ^FAM134B+^ cells revealed that the increase in cytochrome c-dependent apoptosis is the product of FAM134B-mediated ER-phagy. When autophagosome formation exceeds lysosomal degradation capacity, autophagy induces apoptosis [50], which highlights that MAMs are crucial for maintaining the function of this biosynthetic organelle.

It has been reported that mitochondrial apoptosis is induced by Ca ^2+^ overload through the opening of the mitochondrial permeability transition pore on the mitochondrial outer membrane, leading to rapid collapse of the membrane potential and swelling of the mitochondria [51]. Mitochondrial apoptosis reduces the energy supply of cells and impairs cellular functions such as invasion, consequently resulting in impaired embryo implantation and altered fetal development [52]. These findings parallel our observations in the invasion test conducted in our study; inadequate trophoblast invasion into the decidua and poor transformation of the spiral arteries are associated with the pathogenesis of PE. Moreover, excessive mitochondrial Ca ^2+^ is also directly linked to enhanced ROS production [53], and ROS are antiangiogenic factors involved in placental development. Overexpressing FAM134B in HTR-8/SVneo cells in our study resulted in a significant increase in ROS levels, which was reversed by the autophagy inhibitor. ROS accumulation leads to proliferation, the hallmark of arterial stiffness, lipid deposition, vascular remodeling and other vascular pathological changes [ 54, 55] . These findings support the notion that excessive ER-phagy mediated by FAM134B impairs mitochondrial function in trophoblast cells, likely contributing to impaired vascular remodeling ability.

The pathogenesis of PE is associated with a marked decrease in polyunsaturated fatty acids [56]. In this study, GLA was shown to play an important role in mitochondrial apoptosis after FAM134B overexpression. The proposed primary mechanism for apoptosis induction by GLA deficiency is oxidative stress, which is attributed to Ca ^2+^ overload, mitochondrial dysfunction, Ca ^2+^ store depletion [57], ROS accumulation, and lipid peroxidation [58]. Our previous research indicated that polyunsaturated omega-3 and omega-6 fatty acids, including GLA, DHA, and DPA, in mitochondria might be involved in dysregulating PE placentas [59]. In this study, mass spectrometry analyses clearly revealed that FAM134B overexpression resulted in lower concentrations of GLA and fatty acid enzymes, suggesting that fatty acid metabolism might have evolved a specific mechanism to cope with the difficulties associated with mitochondrial apoptosis in PE. We observed significantly lower expressions of Δ5 and Δ6 desaturase, as well as long-chain fatty acid elongase (ELOVL5, which acts on C18 and C20 fatty acids), in FAM134B-overexpressing cells than in control cells, suggesting compromised synthesis downstream of C18. A previous study was consistent with our results, showing that reduced activities of the Δ5 and Δ6 desaturases hindered the formation of GLA precursors, leading to decreased GLA formation [60]. Therefore, excessive ER-phagy may reduce GLA precursor synthesis and thereby lead to the loss of mitochondrial membrane potential and the release of cytochrome c, resulting in mitochondrial apoptosis. In this regard, the alteration in the lipidomic profile of FAM134B-elicited PE strains identified in this study likely points to a new direction in the PE field. Our work highlights that excessive ER-phagy mediated by FAM134B may be related to impaired mitochondrial function, altered fatty acid metabolism and compromised invasion in PE.

In summary, our results demonstrated that FAM134B is essential for maintaining ER-phagy homeostasis. Excessive ER-phagy causes Ca ^2+^ overflow from the ER to mitochondria via MAMs, which results in mitochondrial apoptosis. The latter may be associated with impaired invasion capacity and altered fatty acid metabolism related to PE. Elucidation of the role of FAM134B overexpression in ER-phagy homeostasis and subsequent mitochondrial function further enhances our understanding of PE etiology and provides a potential new therapeutic target for the prevention and treatment of PE.

## Supporting information

23493supplementary_figures

## Supplementary Data

Supplementary data is available at *Acta Biochimica et Biophysica Sinica* online.
