# Corrigendum to: Excessive ER-phagy mediated by FAM134B contributes to trophoblast cell mitochondrial dysfunction in preeclampsia

**DOI:** 10.3724/abbs.2025001

**Published:** 2025-03-25

**Authors:** Andi Wang, Zhuo Li, Dan Zhang, Chang Chen, Hua Zhang

*Acta Biochim Biophys Sin* 2024, 56(10): 1446–1459

https://doi.org/10.3724/abbs.2024065

In the version of this article initially published, an error was found in Figure 1A. The correct figure is as follows, and the correction does not significantly impact the overall findings and conclusions of the paper. The authors apologize for this error and any confusion it may have caused.

**Figure 1:**
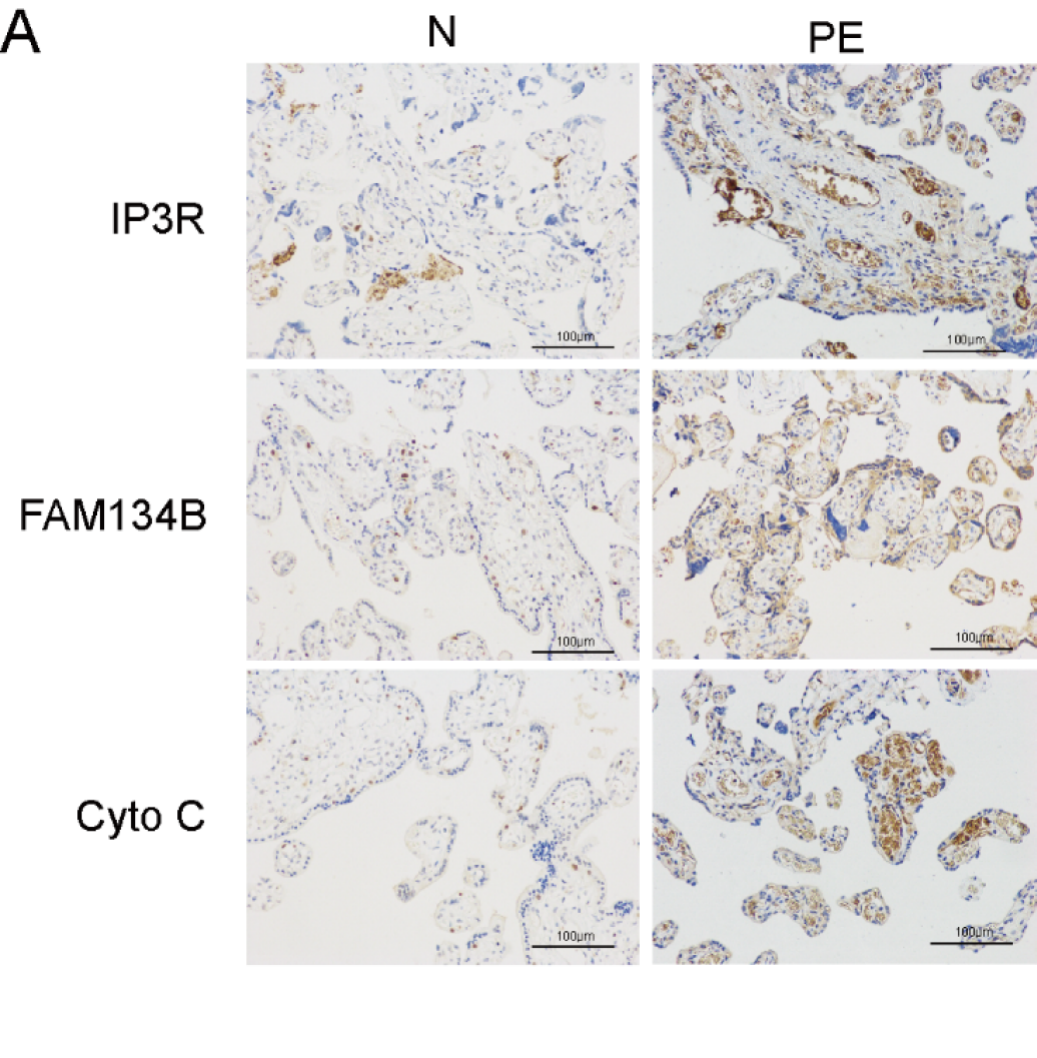
Figure 1A Immunohistochemistry (200×) of FAM134B, IP3R and Cyto C showing the levels in the placentas of normal pregnant women and pregnant women with PE

