# Supplementary material for: Excessive ER-phagy mediated by FAM134B contributes to trophoblast cell mitochondrial dysfunction in preeclampsia: Excessive ER-phagy causes mitochondrial apoptosis in preeclampsia trophoblast
Source: Acta Biochim Biophys Sin (Shanghai). 2024 May 22;56(10):1446–59. doi: 10.3724/abbs.2024065 (PMC11532218; doi:10.3724/abbs.2024065)
Supplement: 23493supplementary_figures [file 23493supplementary_figures.pdf]

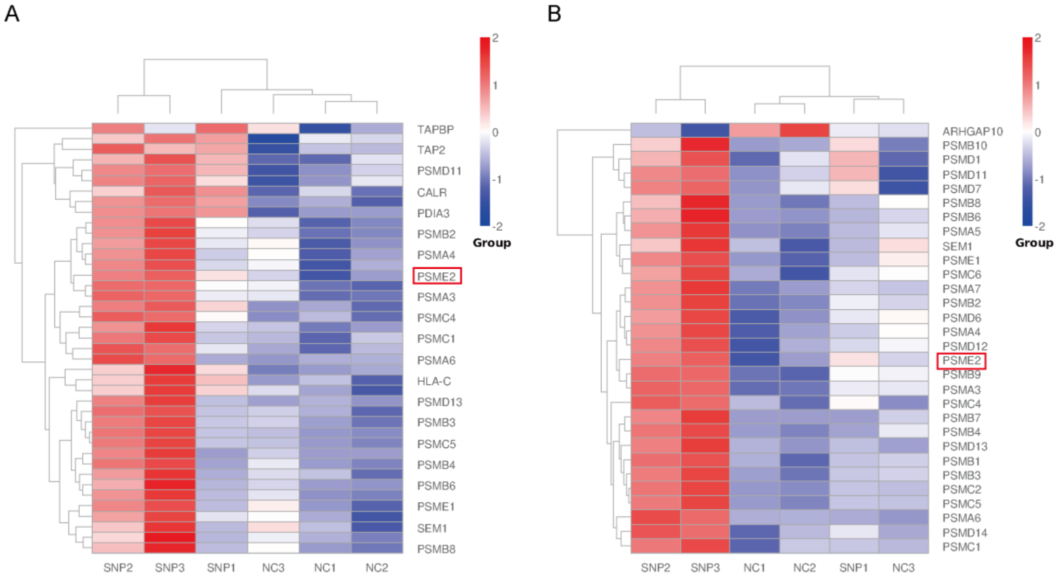

Supplementary Figure S1. Heatmap of RNA-seq data of corresponding genes in the NC group and SNP treatment group (A) Heatmap showing the differences in the ER–phagosome pathway in response to SNP treatment. (B) Heatmap showing the differential gene expression involved in the regulation of apoptosis by SNP treatment. Fold changes in gene expression related to HTR8/SVneo controls are shown in red (increased levels in the SNP treatment group) and blue (decreased levels in the SNP treatment group).

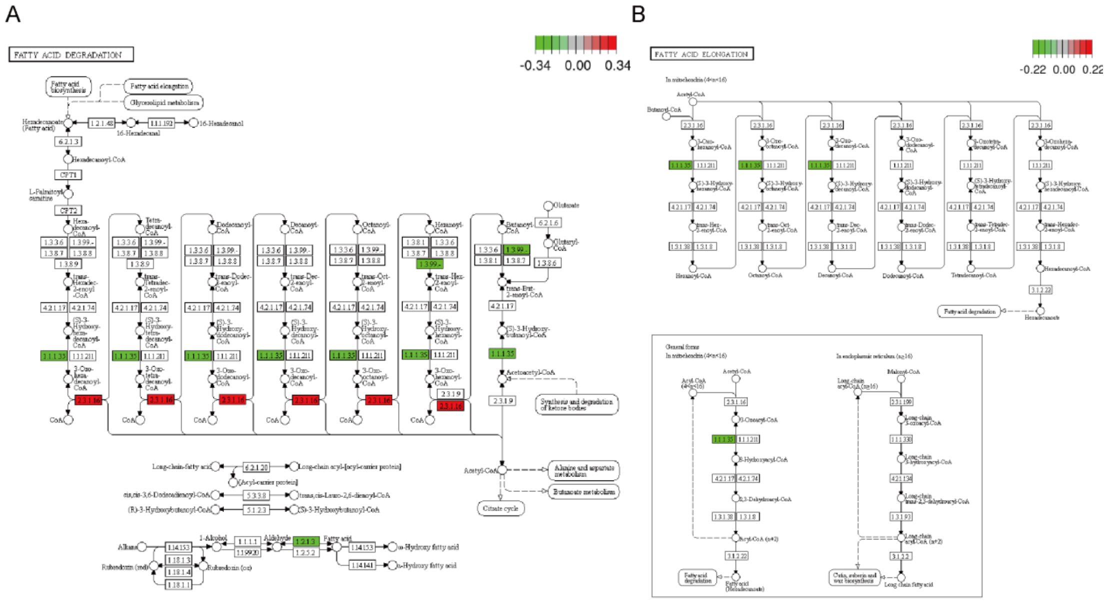

Supplementary Figure S2. Fatty acid metabolism in HTR8/SVneo cells (A) KEGG pathway analysis of fatty acid degradation in HTR8/SVneo cells treated with SNP. (B) KEGG pathway analysis of fatty acid elongation in HTR8/SVneo cells treated with SNP.
